# The high-sensitivity C-reactive protein-to-high-density lipoprotein cholesterol ratio as a novel composite biomarker for predicting 28-day all-cause mortality in sepsis: a retrospective cohort study

**DOI:** 10.3389/fmed.2026.1791886

**Published:** 2026-02-19

**Authors:** Yidong Chen, Wenlin Xie, Liangmei Zhu, Kun Chen, Xinyong Liu

**Affiliations:** 1 Department of Emergency, Affiliated Jinhua Hospital, Zhejiang University School of Medicine, Jinhua, China; 2 Department of Orthopedic Trauma, Affiliated Jinhua Hospital, Zhejiang University School of Medicine, Jinhua, China; 3 Department of Critical Care Medicine, Affiliated Jinhua Hospital, Zhejiang University School of Medicine, Jinhua, China

**Keywords:** HCHR, high-density lipoprotein cholesterol, high-sensitivity C-reactive protein, prognosis, sepsis

## Abstract

**Background:**

Sepsis remains a leading cause of critical illness and mortality, and early risk stratification relies on biomarkers with variable performance. High inflammatory activity and dysregulated lipid metabolism are central features of sepsis, yet the prognostic value of composite inflammatory-metabolic biomarkers remains insufficiently clarified. The high-sensitivity C-reactive protein-to-high-density lipoprotein cholesterol ratio (hs-CRP/HDL-C ratio, HCHR) has been proposed as a novel composite biomarker. HCHR integrates inflammatory and metabolic information, potentially offering superior prognostic insights.

**Methods:**

A total of 1,069 patients with sepsis admitted to the ICU of Affiliated Jinhua Hospital, Zhejiang University School of Medicine between May 2015 and March 2025 were retrospectively enrolled. Patients were divided into quartiles based on HCHR. The primary outcome was 28-day all-cause mortality. Multivariable logistic regression (fully adjusted models) was conducted to assess the association between HCHR and 28-day mortality. Sensitivity analyses excluded lipid-related covariates (TG and LDL-C). Spearman’s partial correlation was performed with pooling across imputations. Robustness was assessed through subgroup analysis and restricted cubic spline (RCS) modeling, while discrimination was evaluated using receiver operating characteristic (ROC) curves with DeLong tests. Internal validation of the fully adjusted model was conducted using 5-fold cross-validation and bootstrap optimism correction.

**Results:**

In the fully adjusted model, higher HCHR was independently associated with increased 28-day mortality (OR 6.10, 95% CI 3.48–10.68, *p* < 0.001). The association remained robust in sensitivity analyses excluding TG and LDL-C (OR 5.40, 95% CI 3.19–9.14). Subgroup analysis revealed a stronger association among patients aged ≥65 years and those with hypertension (*P* for interaction = 0.002 and 0.014, respectively). RCS modeling indicated a linear positive relationship between HCHR and 28-day mortality (*P* for non-linearity = 0.748). HCHR showed moderate discrimination for predicting 28-day mortality (AUC 0.686, 95% CI 0.651–0.722), outperforming hs-CRP and HDL-C alone.

**Conclusion:**

HCHR is independently associated with 28-day all-cause mortality in sepsis and demonstrates moderate discriminative performance. HCHR may serve as a useful adjunct biomarker to support early risk stratification in sepsis.

## 1 Introduction

Sepsis is a life-threatening organ dysfunction triggered by severe infection and remains a major global health challenge due to its persistently high mortality (1, 2). Accurate early risk stratification is essential for improving prognosis, and reliable, easily accessible biomarkers play a critical role in this process (3). In sepsis, two key pathophysiological components dominate: the overwhelming inflammatory cascade initiated by pathogen invasion, and profound metabolic dysregulation (4, 5). Conventional inflammatory biomarkers, such as high-sensitivity C-reactive protein (hs-CRP) and procalcitonin (PCT), broadly reflect infection severity but have limited diagnostic specificity (6). In particular, hs-CRP is frequently elevated in chronic non-infectious conditions (e.g., diabetes, autoimmune disorders, chronic liver disease), thereby reducing its ability to reliably distinguish infectious from non-infectious inflammation (7–9). High-density lipoprotein cholesterol (HDL-C), which exerts anti-inflammatory and antioxidant effects and participates in reverse cholesterol transport, is an important protective molecule in lipid metabolism (10, 11). During sepsis, pro-inflammatory cytokines (e.g., TNF-*α*, IL-6) impair the structure and function of HDL-C and cause a rapid decline in circulating HDL-C levels, which has been linked to poor prognosis in sepsis patients (12, 13). However, HDL-C alone primarily reflects metabolic protection and fails to capture the complex interaction between inflammation and metabolic dysfunction. Despite extensive research on individual inflammatory or lipid biomarkers, the current evidence on composite inflammation-metabolic indices for sepsis prognosis remains limited, and there is no widely accepted composite marker that can robustly capture the interaction between systemic inflammation and metabolic dysregulation in sepsis (14, 15), partly due to small sample sizes, heterogeneity in study populations and outcomes, and limited external validation (14). In recent years, the composite inflammation-metabolic ratio has demonstrated superior prognostic value in cardiovascular diseases (16), but its utility in sepsis remains under-validated.

The hs-CRP/HDL-C ratio (HCHR) constitutes a composite biomarker that integrates both inflammatory injury (elevated hs-CRP) and metabolic protection loss (reduced HDL-C) (17). Across cardiovascular and metabolic disease settings, multiple studies have consistently demonstrated that higher HCHR is associated with worse clinical outcomes, including increased risk of mortality and major adverse events, and in many analyses it shows stronger risk stratification than hs-CRP or HDL-C alone (16, 18, 19). Given that sepsis is fundamentally driven by an interplay between uncontrolled inflammation and metabolic dysregulation, we propose that HCHR may serve as a more accurate prognostic indicator in sepsis patients. Therefore, the present study aims to investigate the association between HCHR and 28-day all-cause mortality in a large retrospective cohort of sepsis patients.

## 2 Methods

### 2.1 Study population

**Figure 1:**
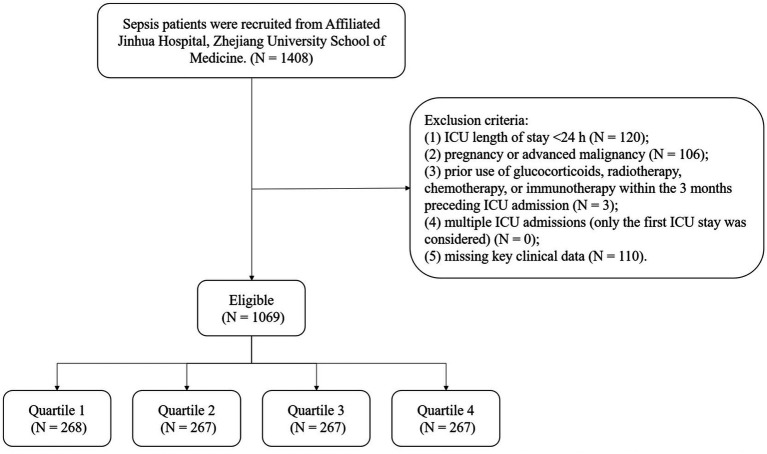
Study flowchart. Flow diagram illustrating patient selection from Affiliated Jinhua Hospital, Zhejiang University School of Medicine, exclusion criteria, and final cohort stratification by hs-CRP/HDL-C ratio (HCHR) quartiles (Q1–Q4).

We retrospectively included 1,069 adult patients with sepsis admitted to the ICU of Affiliated Jinhua Hospital, Zhejiang University School of Medicine between May 2015 and March 2025. Inclusion criteria were as follows (Figure 1): (1) age ≥18 years; (2) diagnosis consistent with Sepsis-3.0 criteria, defined as a suspected or confirmed infection with an acute increase in SOFA score of ≥2 points. Confirmed or suspected infection was determined by reviewing clinical symptoms/signs, imaging findings, inflammatory laboratory markers, and microbiological cultures (e.g., blood, sputum, urine, feces, and other relevant body fluids) documented in the medical record. The SOFA score was calculated using the worst values of each component recorded within the first 24 h after ICU admission; (3) sepsis diagnosis within 24 h before or after ICU admission. Exclusion criteria were: (1) ICU length of stay <24 h; (2) pregnancy or advanced malignancy; (3) prior use of glucocorticoids, radiotherapy, chemotherapy, or immunotherapy within the 3 months preceding ICU admission; (4) multiple ICU admissions (only the first ICU stay was considered); (5) missing key clinical data.

### 2.2 Data collection

Clinical data within the first 24 h of ICU admission were extracted from the electronic medical record system, including: (1) demographics: age, gender, smoking status, and drinking status; (2) comorbidities: hypertension, diabetes, chronic heart disease, and chronic pulmonary disease; (3) clinical variables: need for mechanical ventilation, and mean arterial pressure (MAP); (4) laboratory variables (worst values within 24 h): leukocytes, neutrophils, lymphocytes, monocytes, alanine aminotransferase (ALT), aspartate aminotransferase (AST), albumin, creatinine, lactate, hs-CRP, total cholesterol (TC), triglycerides (TG), HDL-C, and low-density lipoprotein cholesterol (LDL-C). HCHR = hs-CRP (mg/L)/HDL-C (mmol/L) (17). Patients were categorized into quartiles according to HCHR. The primary outcome was 28-day all-cause mortality.

### 2.3 Statistical analysis

Statistical analyses were performed using R software (version 4.4.3). Missing values <10% were handled using multiple imputation generated by the MICE package. Five imputed datasets were generated (*m* = 5) with 10 iterations each, using predictive mean matching (PMM). The following variables were imputed: MAP, ALT, AST, albumin, creatinine, lactate, and LDL-C. The 28-day mortality and HCHR were excluded from imputation. Normality of continuous variables was assessed using the Shapiro–Wilk test. Continuous variables were compared using *t*-tests (normally distributed; expressed as mean ± SD) or Wilcoxon rank-sum tests (non-normally distributed; expressed as median [Q1, Q3]). Categorical variables were compared using chi-square or Fisher’s exact tests (expressed as *n*, %). Variance inflation factor (VIF) > 5 was considered evidence of multicollinearity. No evidence of problematic multicollinearity was observed (all VIFs < 2).

To evaluate the association between HCHR and 28-day mortality, three logistic regression models were constructed: (1) Model 1: unadjusted; (2) Model 2: adjusted for age and gender; (3) Model 3: adjusted for age, gender, diabetes, chronic pulmonary disease, mechanical ventilation, MAP, neutrophils, lymphocytes, monocytes, albumin, creatinine, TG, LDL-C, and lactate. Given that sepsis is frequently accompanied by broad dyslipidemia and metabolic disorders, and that lipid parameters may be associated with disease severity and mortality. Including TG and LDL-C as covariates aimed to examine whether the association between HCHR and 28-day mortality extended beyond general lipid abnormalities. To address the possibility of overadjustment due to correlations with HDL-C and other lipid parameters (TG, LDL-C), we further performed sensitivity analyses excluding lipid-related covariates (TG and LDL-C). Additionally, Spearman’s partial correlation was used to assess the association between HCHR and 28-day mortality status after adjustment for covariates, with pooling across imputations.

Moreover, subgroup analyses were performed by stratifying patients based on age, gender, smoking status, drinking status, hypertension, diabetes, chronic heart disease, chronic pulmonary disease, and mechanical ventilation. Interaction *p*-values were calculated. Restricted cubic spline (RCS) analyses were used to explore potential non-linear associations. Continuous variables included in regression models, subgroup analyses, and restricted cubic spline analyses were standardized using z-score normalization prior to model fitting to improve comparability and model stability. Receiver operating characteristic (ROC) curves were constructed to evaluate predictive performance, and AUCs were compared using the DeLong test. To enhance credibility and reproducibility, internal validation of Model 3 was performed using fixed stratified 5-fold cross-validation and bootstrap optimism correction, and discrimination was summarized by the cross-validated AUC and the bootstrap optimism-corrected AUC (95% CI). *p* value < 0.05 was considered statistically significant.

## 3 Results

### 3.1 Baseline characteristics

Baseline characteristics across HCHR quartiles revealed a clear severity gradient. Compared with patients in the lowest quartile (Q1), those in the highest quartile (Q4) exhibited more severe clinical profiles, including higher prevalence of diabetes, greater need for mechanical ventilation, and more abdominal infections (*p* < 0.05). Laboratory findings in Q4 patients revealed elevated TG, hs-CRP, and creatinine, along with reduced MAP, albumin, and HDL-C (*p* < 0.05). Correspondingly, 28-day mortality increased progressively across quartiles (*p* < 0.05). No significant differences were observed for age or gender (*p* > 0.05) (Table 1).

**Table 1:** Patient’s clinical characteristics according to HCHR quartiles.

Variables	Total (1,069)	Q1 (268)	Q2 (267)	Q3 (267)	Q4 (267)	*P*
Gender						0.393
Male, *n* (%)	693 (64.8)	174 (64.9)	178 (66.7)	162 (60.7)	179 (67.0)	
Female, *n* (%)	376 (35.2)	94 (35.1)	89 (33.3)	105 (39.3)	88 (33.0)	
Age, years	70.0 [58.0, 80.0]	69.5 [56.0, 79.0]	72.0 [58.0, 80.5]	71.0 [58.5, 79.0]	69.0 [57.5, 80.5]	0.463
Smoking status, *n* (%)	388 (36.3)	101 (37.7)	106 (39.7)	85 (31.8)	96 (36.0)	0.275
Drinking status, *n* (%)	345 (32.3)	95 (35.4)	82 (30.7)	75 (28.1)	93 (34.8)	0.215
Comorbidities						
Hypertension, *n* (%)	525 (49.1)	145 (54.1)	125 (46.8)	119 (44.6)	136 (50.9)	0.122
Diabetes, *n* (%)	301 (28.2)	71 (26.5)	61 (22.8)	80 (30.0)	89 (33.3)	**0.045**
Chronic heart disease, *n* (%)	306 (28.6)	86 (32.1)	82 (30.7)	68 (25.5)	70 (26.2)	0.24
Chronic pulmonary disease, *n* (%)	209 (19.6)	55 (20.5)	70 (26.2)	48 (18.0)	36 (13.5)	**0.002**
Mechanical ventilation, *n* (%)	638 (59.7)	167 (62.3)	146 (54.7)	142 (53.2)	183 (68.5)	**0.001**
MAP, mmHg	85.0 [72.0, 98.0]	88.0 [73.0, 102.0]	86.0 [73.0, 100.5]	84.0 [71.0, 98.0]	82.0 [71.0, 93.0]	**0.005**
Laboratory parameters						
Leukocytes, 10^9^/L	13.7 [9.0, 20.1]	12.4 [8.6, 17.8]	14.1 [10.0, 20.3]	13.7 [9.0, 21.0]	13.8 [8.8, 21.4]	0.062
Neutrophils, 10^9^/L	12.1 [7.8, 18.3]	10.7 [7.1, 16.2]	12.4 [8.4, 18.6]	12.6 [8.1, 18.6]	12.6 [7.7, 19.4]	**0.019**
Lymphocytes, 10^9^/L	0.5 [0.3, 0.8]	0.6 [0.4, 1.0]	0.5 [0.3, 0.8]	0.5 [0.3, 0.7]	0.5 [0.3, 0.8]	**<0.001**
Monocytes, 10^9^/L	0.6 [0.3, 0.9]	0.6 [0.4, 1.0]	0.6 [0.4, 0.9]	0.5 [0.3, 0.8]	0.5 [0.2, 0.8]	**<0.001**
ALT, U/L	36.9 [22.0, 90.4]	36.0 [20.0, 101.6]	36.0 [21.6, 81.8]	36.4 [20.6, 96.8]	40.0 [22.9, 86.0]	0.905
AST, U/L	60.5 [34.0, 164.0]	63.8 [32.0, 204.5]	52.0 [31.5, 128.8]	58.6 [35.0, 161.5]	70.9 [39.5, 163.5]	0.056
Albumin, g/L	27.0 [24.2, 30.3]	30.5 [27.2, 34.0]	27.6 [25.0, 30.4]	26.8 [24.0, 29.1]	24.5 [21.4, 26.8]	**<0.001**
Creatinine, μmol/L	132.0 [85.0, 252.0]	117.0 [77.0, 221.6]	118.0 [87.7, 207.4]	127.5 [84.0, 260.0]	190.0 [106.0, 317.0]	**<0.001**
Lactate, mmol/L	2.3 [1.5, 4.2]	2.2 [1.5, 4.6]	2.0 [1.3, 3.5]	2.2 [1.5, 4.1]	2.6 [1.7, 4.8]	**<0.001**
hs-CRP, mg/L	123.9 [63.3, 177.2]	29.8 [14.2, 51.4]	109.1 [82.4, 145.1]	159.5 [121.2, 191.3]	182.2 [155.8, 200.0]	**<0.001**
TC, mmol/L	2.8 [2.1, 3.5]	3.4 [2.7, 4.2]	3.0 [2.4, 3.6]	2.6 [2.1, 3.2]	2.1 [1.7, 2.8]	**<0.001**
TG, mmol/L	1.3 [0.9, 1.9]	1.1 [0.8, 1.6]	1.1 [0.8, 1.7]	1.4 [1.0, 1.9]	1.6 [1.2, 2.4]	**<0.001**
HDL-C, mmol/L	0.8 [0.5, 1.0]	1.0 [0.8, 1.2]	0.9 [0.7, 1.2]	0.8 [0.6, 0.9]	0.5 [0.3, 0.6]	**<0.001**
LDL-C, mmol/L	1.7 [1.3, 2.2]	2.1 [1.6, 2.7]	1.8 [1.3, 2.3]	1.6 [1.2, 2.1]	1.5 [1.1, 1.9]	**<0.001**
HCHR (ratio)	158.3 [78.9, 267.9]	34.0 [15.5, 55.4]	121.9 [100.9, 143.0]	206.2 [184.4, 235.3]	386.7 [312.5, 512.8]	**<0.001**
28-day all-cause mortality, *n* (%)	340 (31.8)	52 (19.4)	56 (21.0)	83 (31.1)	149 (55.8)	**<0.001**

ALT, Alanine Aminotransferase; AST, Aspartate Aminotransferase; hs-CRP, High-sensitivity C-reactive Protein; TC, Total Cholesterol; TG, Triglycerides; HDL-C, High-Density Lipoprotein Cholesterol; LDL-C, Low-Density Lipoprotein Cholesterol; HCHR, hs-CRP/HDL-C ratio. Values in bold indicate statistical significance (*P* < 0.05).

### 3.2 Association between HCHR and 28-day mortality

Multivariate logistic regression analysis was performed to examine the association between HCHR quartiles and 28-day all-cause mortality in sepsis patients, using the lowest HCHR quartile (Q1) as the reference group. In the unadjusted model (Model 1), both Q3 and Q4 were associated with higher mortality (Q3: OR 1.87, 95% CI 1.26–2.79, *p* = 0.002; Q4: OR 5.25, 95% CI 3.56–7.73, *p* < 0.001). After adjusting for age and gender (Model 2), the association for Q4 remained significant (OR 5.29, 95% CI 3.57–7.83, *p* < 0.001). In the fully adjusted model (Model 3), which accounted for additional potential confounders, the independent associations of Q3 and Q4 with mortality persisted, with Q4 showing a markedly higher risk compared to Q1 (OR 6.10, 95% CI 3.48–10.68, *p* < 0.001). Q2 was not significantly associated with mortality in any model (Table 2). In sensitivity analyses excluding lipid-related covariates (TG and LDL-C), the association between HCHR and 28-day mortality remained robust. Compared with Q1, patients in Q4 had an adjusted OR of 5.40 (95% CI 3.19–9.14) for 28-day mortality (Supplementary Table S1). Additionally, Spearman’s partial correlation analysis showed that HCHR remained positively correlated with 28-day mortality status after adjustment for the covariates in Model 3 (pooled partial *ρ* = 0.253, 95% CI 0.196–0.310, *p* < 0.001).

**Table 2:** Relationship between HCHR and 28-day mortality in septic patients.

HCHR quartiles	Model 1		Model 2		Model 3	
	OR (95% CI)	*P*	OR (95% CI)	*P*	OR (95% CI)	*P*
Q1	1		1		1	
Q2	1.10 (0.72–1.68)	0.651	1.05 (0.69–1.61)	0.817	1.26 (0.76–2.08)	0.378
Q3	1.87 (1.26–2.79)	0.002	1.86 (1.24–2.78)	0.003	2.42 (1.44–4.07)	<0.001
Q4	5.25 (3.56–7.73)	<0.001	5.29 (3.57–7.83)	<0.001	6.10 (3.48–10.68)	<0.001

Model 1: no covariates were adjusted.

Model 2: age and gender were adjusted.

Model 3: age, gender, diabetes, Chronic pulmonary disease, mechanical ventilation, MAP, neutrophils lymphocytes, monocytes, albumin, creatinine, TG, LDL-C, lactate.

### 3.3 Subgroup analysis

**Figure 2:**
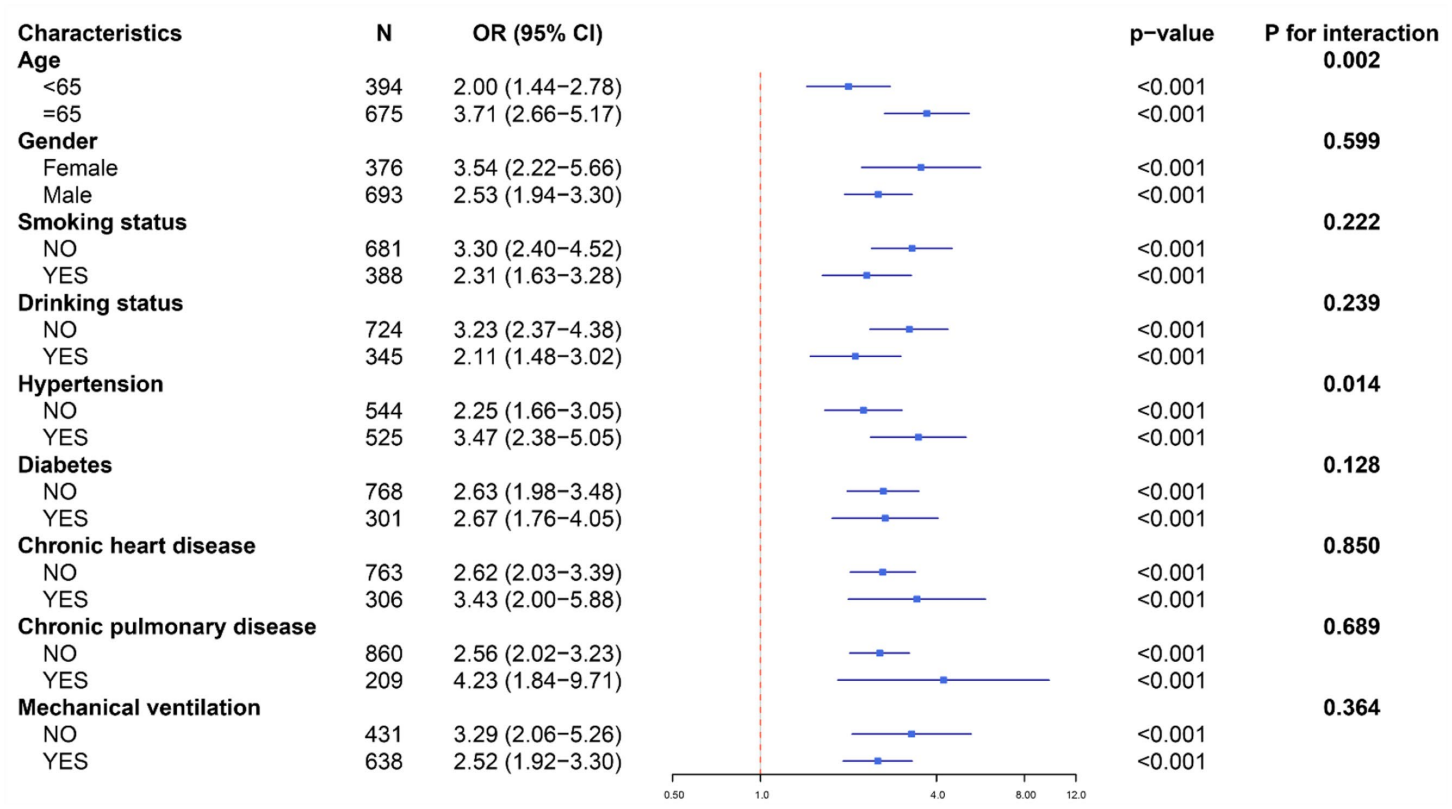
Subgroup analyses examining the association between HCHR and 28-day mortality.

Subgroup analyses stratified by age, gender, smoking status, drinking status, hypertension, diabetes, chronic heart disease, chronic pulmonary disease, and mechanical ventilation consistently indicated that HCHR was associated with higher 28-day mortality in sepsis patients (all *p* < 0.001). Significant interactions were observed for age (≥65 years; *P* for interaction = 0.002) and hypertension (*P* for interaction = 0.014), with stronger associations in these subgroups. No substantial interactions were detected in the remaining subgroups (Figure 2).

### 3.4 Non-linear relationship analysis

**Figure 3:**
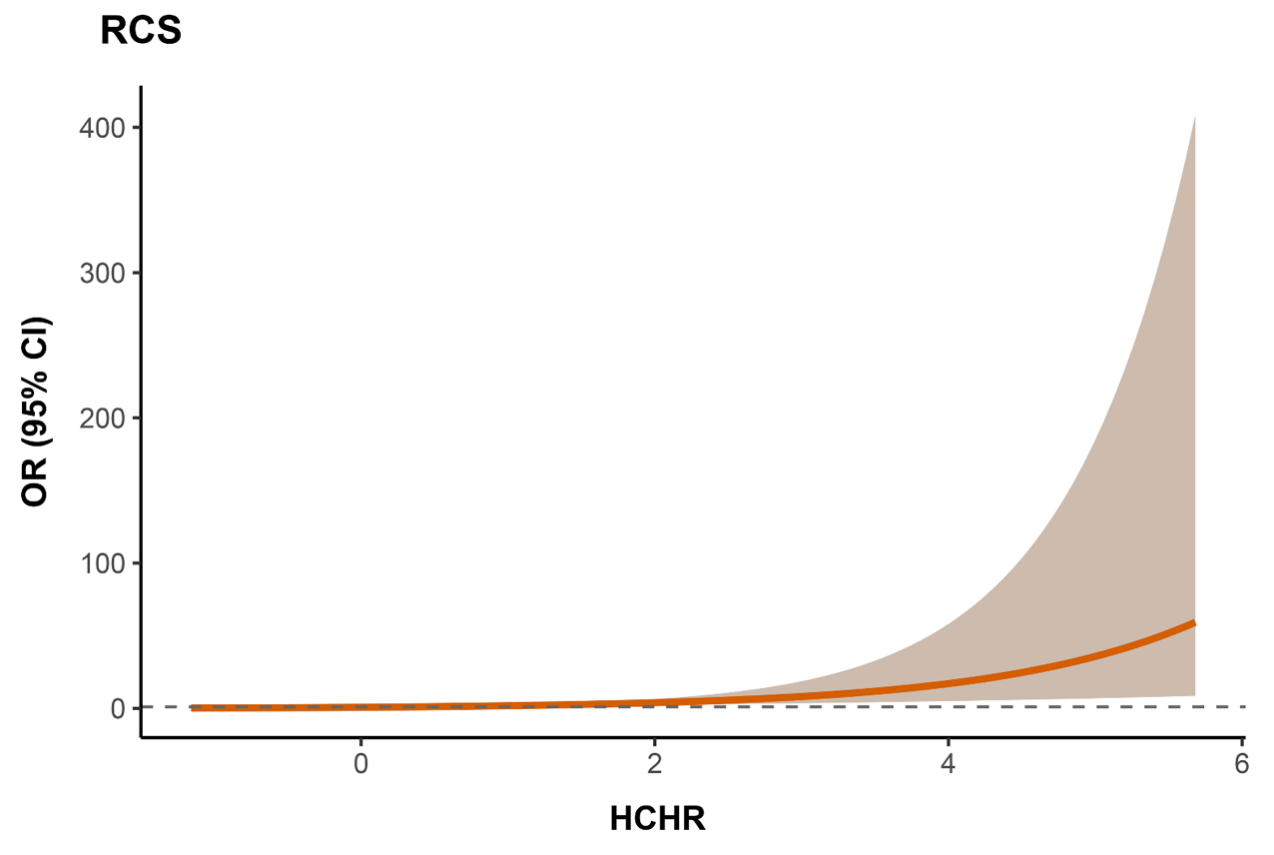
Association between HCHR and 28-day mortality using restricted cubic spline (RCS) analysis.

RCS analysis revealed a linear, positive association between HCHR and 28-day mortality (*P* total < 0.001). The risk of 28-day mortality increased steadily with higher HCHR, with no evidence of nonlinear relationship (P for non-linearity = 0.748) (Figure 3). These findings suggest that HCHR may serve as a continuous risk marker in clinical practice rather than a threshold-based indicator.

### 3.5 Predictive performance of HCHR

**Figure 4:**
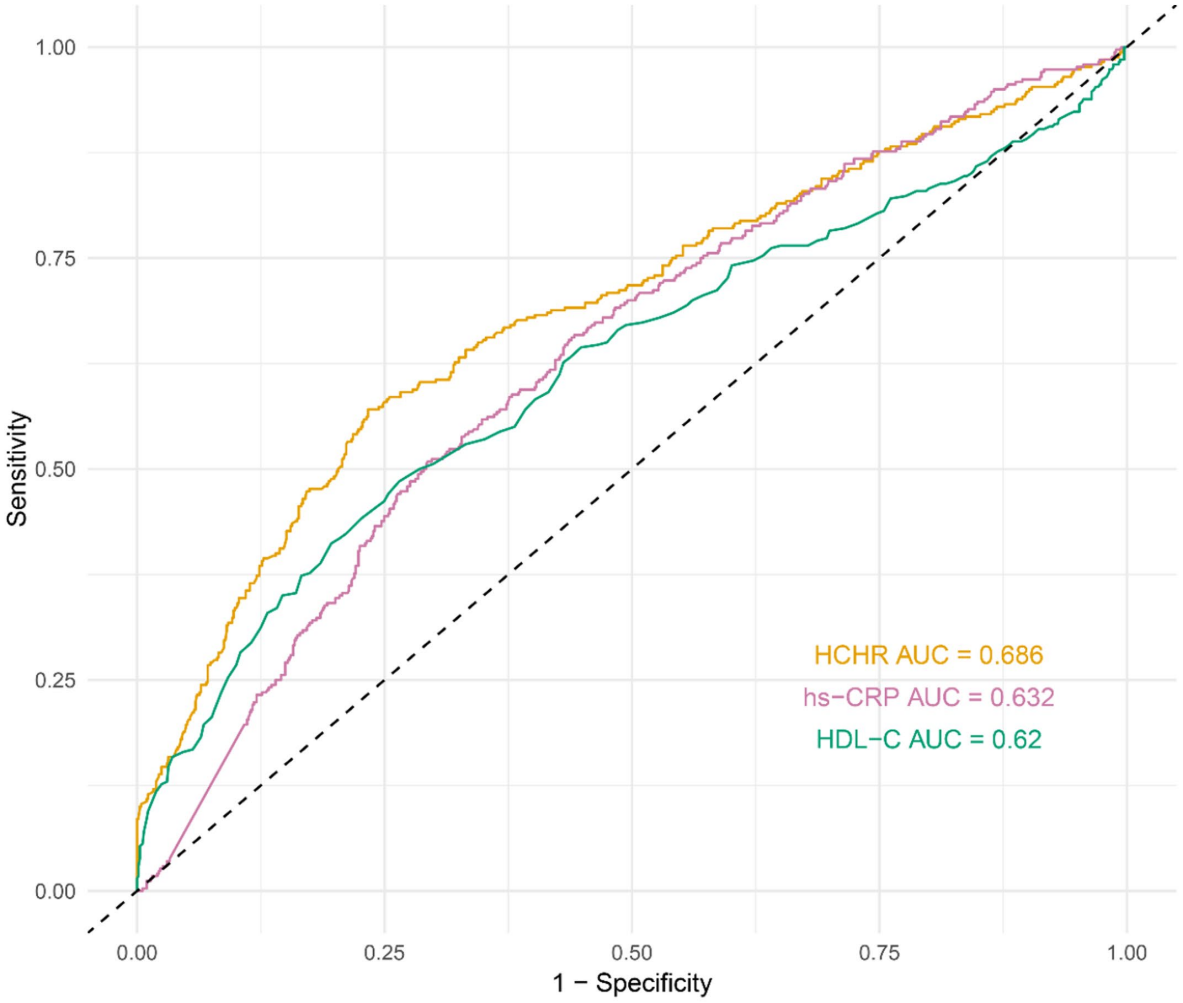
The ROC curve of HCHR in predicting 28-day mortality in septic patients.

ROC curve analysis demonstrated that HCHR effectively predicted 28-day mortality, with an AUC of 0.686 (95% CI 0.651–0.722), outperforming both hs-CRP (AUC 0.632, 95% CI 0.596–0.667) and HDL-C (AUC 0.620, 95% CI 0.582–0.659) (*p* < 0.05) (Figure 4). The DeLong test showed that the AUC for HCHR was significantly higher than that for hs-CRP and HDL-C (*p* < 0.05). To further evaluate the robustness of discrimination for the fully adjusted multivariable model (Model 3), internal validation was performed. Internal validation demonstrated stable discrimination. The mean AUC from 5-fold cross-validation was 0.837 (95% CI 0.825–0.853; between-imputation SD = 0.0017), and the bootstrap optimism-corrected AUC was 0.834 (95% CI 0.810–0.858; between-imputation SD = 0.0018), indicating minimal optimism. Overall, the AUC indicated moderate discrimination, supporting the use of HCHR for risk stratification rather than standalone decision-making.

## 4 Discussion

This study investigated the utility of HCHR as an integrated inflammatory–metabolic biomarker for prognostic assessment in sepsis. In this large retrospective cohort, higher HCHR was independently associated with increased 28-day all-cause mortality after adjustment for demographic characteristics, comorbidities, clinical variables, and laboratory parameters (fully adjusted model: OR 6.10, 95% CI 3.48–10.68). Moreover, HCHR demonstrated moderate discrimination (AUC 0.686), outperforming hs-CRP or HDL-C alone, suggesting that combining inflammatory burden with metabolism may provide more informative risk stratification than single biomarkers.

The pathophysiological basis of HCHR in sepsis lies in its capture of overwhelming systemic inflammatory and lipid metabolic defense mechanisms. First, hs-CRP is a downstream effector largely driven by IL-6 signaling and reflects the magnitude of systemic inflammatory activation (20). This inflammatory surge contributes to endothelial dysfunction, microcirculatory derangements, and organ injury (20), which are central to sepsis-related mortality. However, because hs-CRP can also increase in various chronic inflammatory conditions, its specificity for assessing infection alone is limited (7–9). Second, decreased HDL-C is not merely a marker of dyslipidemia but reflects the collapse of key homeostatic systems. HDL-C exerts pleiotropic protective effects relevant to sepsis, including endotoxin neutralization, anti-inflammatory and anti-oxidative actions, modulation of innate immune responses, and preservation of endothelial barrier integrity and microvascular function (21, 22). During sepsis, inflammatory cytokines suppress HDL-C synthetase activity and disrupt HDL-C structure, leading to decreased levels and functional inactivation (23). Reduced HDL-C further impairs the body’s compensatory capacity against inflammation, forming a vicious cycle of exacerbated inflammation and weakened protection (24). HCHR quantifies the degree of inflammatory injury via elevated hs-CRP and the loss of metabolic protective capacity via reduced HDL-C. This ratio serves as a composite indicator of the injury-protection imbalance in sepsis. A heightened ratio reflects greater inflammatory severity and inadequate metabolic compensation, both of which are associated with an unfavorable prognosis. This plausibly explains why HCHR provides more discriminative prognostic information than either component alone. Previous studies have supported this: Gao et al. demonstrated in a large national cohort that HCHR exhibited a significantly higher AUC for predicting cardiovascular events in middle-aged and elderly individuals compared with hs-CRP or lipid markers alone (16). Sun et al., using data from NHANES 2015–2023, further reported that HCHR was positively associated with the risk of prediabetes and diabetes, suggesting its utility in early metabolic risk stratification (19). In addition, Chen et al. found that elevated preoperative HCHR increased the likelihood of postoperative SIRS in elderly patients (18). Our study also validated the clinical utility of HCHR in a large-sample sepsis population.

Our results are also consistent with existing clinical literature on the individual components of HCHR in sepsis and critical illness. Multiple studies have demonstrated that elevated CRP or hs-CRP independently predicts mortality in septic or critically ill populations (25, 26), whereas reduced HDL-C levels correlate with heightened cytokine release, increased vasopressor demand, and poor survival outcomes (23, 27). More recently, composite biomarkers combining inflammatory and lipid-related parameters have been shown to outperform single indices in risk stratification, further corroborating the clinical relevance of integrative markers such as HCHR (16, 18, 19). Notably, in cardiovascular disease and related metabolic conditions, HCHR has been repeatedly associated with higher risks of major adverse events and mortality, and may provide incremental prognostic value beyond isolated inflammatory or lipid markers, which is consistent with our study findings. The consistency across disease domains supports that HCHR reflects the comprehensive role of systemic inflammation and lipid metabolism (16, 28), which is relevant not only in atherosclerotic cardiovascular disease but also in sepsis, where systemic inflammation, endothelial injury, and metabolic reprogramming coexist (29, 30).

From a clinical perspective, our RCS analysis revealed a linear dose–response relationship between HCHR and 28-day mortality without an apparent threshold, indicating that mortality risk increases progressively with rising HCHR values. Given its moderate discrimination, HCHR should be positioned as an adjunctive tool to support early risk stratification rather than a standalone decision-making biomarker. In practice, several clinical application scenarios merit prospective evaluation. First, HCHR could be incorporated into early triage workflows within the first 24 h of ICU admission to identify patients who may benefit from closer monitoring or intensified reassessment. Second, regarding integration into clinical scoring systems, our findings support considering HCHR as a candidate variable to be added to established prognostic models (e.g., SOFA- or APACHE-based frameworks) to test whether it provides incremental predictive value. But this still needs multicenter prospective verification. Third, dynamic monitoring of HCHR trajectories may offer additional prognostic information beyond a single early measurement, potentially reflecting treatment response and evolving immunometabolic status during hospitalization.

Nevertheless, several limitations should be acknowledged. First, its single-center retrospective design may introduce selection bias. Causal inference cannot be established and the observed associations should not be interpreted as causal effects. Multicenter prospective studies are needed. Second, HCHR was derived from a single early time-point measurement (worst values within 24 h of ICU admission), and the prognostic significance of longitudinal changes and trajectories of HCHR remains unclear. Third, medication use and treatments that may influence lipid profiles and inflammatory markers (e.g., statins, corticosteroids, and lipid-modifying therapies) were not fully captured or adjusted for, which may have affected HDL-C, hs-CRP, and thus HCHR, contributing to residual confounding. Finally, the biological mechanisms by which HCHR affects sepsis prognosis require further experimental exploration.

In summary, HCHR is independently associated with 28-day mortality in sepsis and demonstrates better prognostic discrimination than hs-CRP or HDL-C alone. Given its accessibility and low cost, HCHR may serve as a practical adjunct biomarker for early risk stratification and as a candidate component for future enhanced prognostic models, pending prospective validation.

## Data Availability

The original contributions presented in the study are included in the article/Supplementary material, further inquiries can be directed to the corresponding author.
